# Epstein–Barr Virus Primary Infection Complicated by Hemophagocytic Lymphohistiocytosis and Plasmablastic Lymphoma in a HIV-Negative Patient

**DOI:** 10.1155/2019/7962485

**Published:** 2019-10-07

**Authors:** Nan Chen, Mike Perez, Martha Mims

**Affiliations:** Baylor College of Medicine, 1 Baylor Plaza, Houston, TX 77030, USA

## Abstract

EBV (Epstein–Barr virus) viremia causes immune dysregulation through various mechanisms, and we are understanding more that mutations in B, T, and NK (natural killer) cell signaling pathways allow EBV complications such as HLH (hemophagocytic lymphohistiocytosis) and lymphomas to arise. Here, we report a 20-year-old previously healthy, HIV- (human immunodeficiency virus-) negative male who presented with fevers, sore throat, and lymphadenopathy (LAD). He was found to have EBV viremia, pancytopenia, and elevated LFTs (liver function tests) suspicious for HLH. Bone marrow biopsy and elevated IL-2 (interleukin) receptor confirmed this diagnosis. Additionally, gastric biopsy confirmed diagnosis of plasmablastic lymphoma (PBL), a rare, aggressive HIV- and EBV-associated lymphoma. Both bone marrow and gastric biopsy showed evidence of EBV. Patients with EBV complications should have a rigorous workup to characterize the full extent of immune dysregulation including genetic testing at a high-volume center.

## 1. Case Presentation

The patient was a healthy 20-year-old M who presented with 3 weeks of intermittent fever, sore throat, and painful bilateral cervical lymph nodes. He went to an outside emergency room 5 days prior to presentation and was diagnosed with infectious mononucleosis via positive monospot and discharged with symptomatic treatment. He presented to our institution for 2 days of hematemesis, melena, jaundice, and continued fevers. On interview with his family, he had a healthy childhood without prior hospitalizations and one healthy younger male sibling.

**Figure 1:**
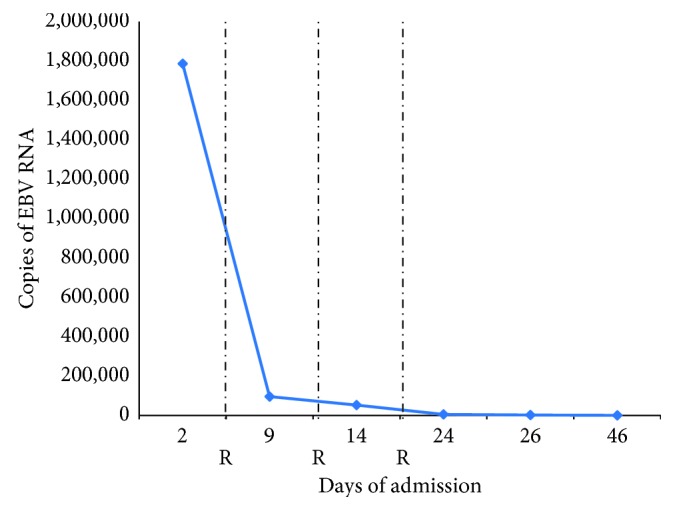
Epstein–Barr virus (EBV) viremia through days of admission. R = Rituximab administration at 375 mg/m^2^.

On physical exam, he was febrile and tachycardic. He had bilateral cervical lymphadenopathy, decreased breath sounds in his bilateral lung bases, and hepatosplenomegaly. Initial laboratory testing showed the following: Hgb (hemoglobin) 12.3 g/dL, WBC (white blood cell) 5.2 k/uL, platelets 68 k/uL, aspartate aminotransferase 361 U/L, alanine aminotransferase 242 U/L, alkaline phosphatase 332 U/L, total bilirubin 10.7 mg/dL, prothrombin time 34.3 seconds, fibrinogen 95 mg/dL, and d-dimer 6.5 ug/mL. HIV (human immunodeficiency virus) and hepatitis panel were negative. CT (computed tomography) of chest, abdomen, and pelvis showed diffuse lymphadenopathy in axillary, mediastinal, hilar, retroperitoneal, and inguinal regions, numerous pulmonary nodules bilaterally, and hepatosplenomegaly. Over the following 24 hours, the patient's clinical condition deteriorated, and the following day, he was intubated for hypoxemic respiratory failure, started on broad-spectrum antibiotics, and given supportive transfusions. EBV viremia was confirmed with a viral load of 2 million copies/mL (Figure 1).

**Figure 2:**
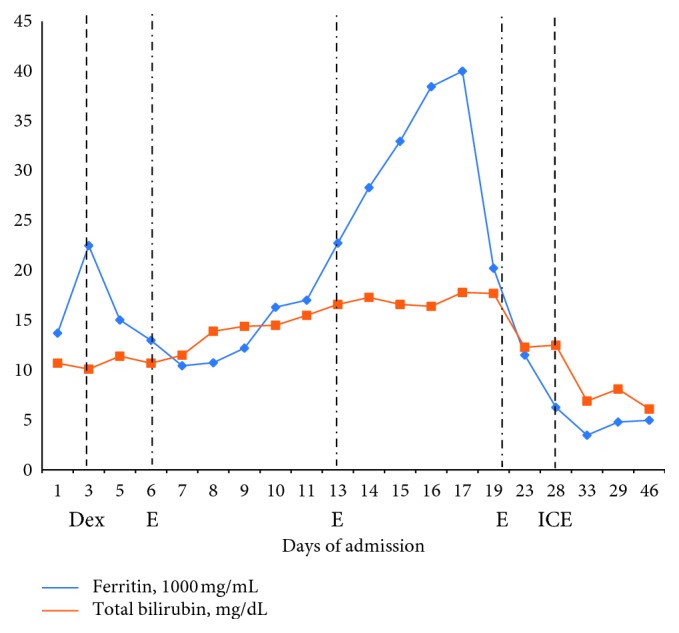
Ferritin and total bilirubin throughout hospital course. Dex = dexamethasone administration; E = etoposide administration; ICE = ifosfamide, carboplatin, and etoposide administration.

An EGD (esophagogastroduodenoscopy) was performed for bleeding, revealing multiple friable, superficial ulcers throughout the distal esophagus and stomach inconsistent with peptic ulcer disease, and biopsies were collected. With dropping blood counts, triglycerides of 289 mg/dL and ferritin of 13,000 ng/mL (Figure 2), there was concern for hemophagocytic lymphohistiocytosis (HLH), and a marrow exam was performed along with a left inguinal lymph node biopsy. Marrow demonstrated “areas with increased macrophages associated with hemophagocytosis and focal necrosis,” consistent with HLH. Inguinal lymph node biopsy also showed hemophagocytosis but was uninvolved by lymphoma. The patient was started on dose-reduced dexamethasone and etoposide (for renal and hepatotoxicity) as per the HLH-94 protocol as well as IVIG (intravenous immunoglobulin) [1]. By day 8, despite numerous supportive transfusions and therapy, laboratory testing showed WBC 1.6 K/uL, Hgb 7.8 g/dL, platelets 13 K/uL, and fibrinogen 89 mg/dL. During this time, his ferritin rose to 28,000 ng/mL. Liver transaminases continued to rise and in conjunction with other laboratory values reflected acute liver failure. Serum immunoglobulins were low. His IL-2 (interleukin) soluble receptor sent earlier in admission returned at 36,000 U/mL (reference range 406–1100 U/mL).

Weekly rituximab was started for EBV viremia. On day 10 of hospital admission, gastric biopsies returned showing a neoplastic infiltrate positive for CD138, CD45, CD79a, CD43, BCL-2, and MUM-1 and negative for CD20 consistent with plasmablastic lymphoma (PBL). Both bone marrow and gastric biopsies were positive for EBER (Epstein–Barr virus-encoded small ribonucleic acids). Initiation of chemotherapy was held, while both HLH treatment and antibiotics for enterococcus bacteremia were ongoing. The patient remained with liver failure, disseminated intravascular coagulation (DIC) requiring daily supportive transfusions, and kidney failure requiring hemodialysis. The patient improved and by day 20 was extubated; EBV viral load decreased to 900 copies/mL, ferritin decreased to 7,000 ng/mL, and pancytopenia improved. Eventually, his blood cultures cleared, and on day 27 of his hospital admission, dose-reduced ifosfamide, carboplatin, and etoposide (ICE) was initiated. Adriamycin was omitted because of low ejection fraction in the setting of acute illness. He tolerated Cycle #1 without any immediate complications. However, 5 days after chemotherapy, despite improving coagulopathy, the patient reported abdominal pain and imaging revealed a nontraumatic, spontaneous retroperitoneal hematoma which was unable to be safely evacuated. Seven days after chemotherapy, he developed worsening pancytopenia and transaminitis, thought to be secondary to ICE as opposed to recurrent HLH. The patient developed neutropenic fever and septic shock, antibiotics were restarted, and CT chest showed right lower lobe pneumonia. Again, he went into respiratory failure, was reintubated, and on bronchoscopy, blood was seen in all lobes of the right lung, consistent with diffuse alveolar hemorrhage. The patient became comfort care and subsequently died.

## 2. Discussion

**Figure 3:**
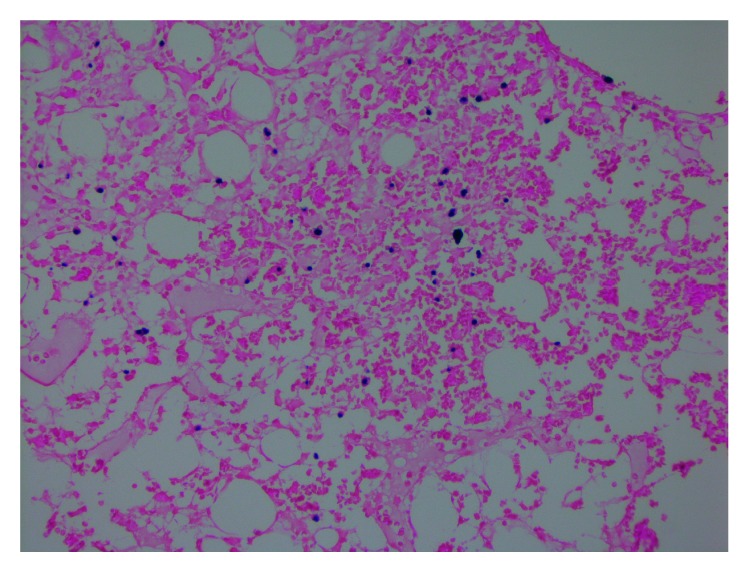
40x of the EBER-ISH (EBV-encoded RNA-in situ hybridization) stain demonstrating EBV activity in the bone marrow.

**Figure 4:**
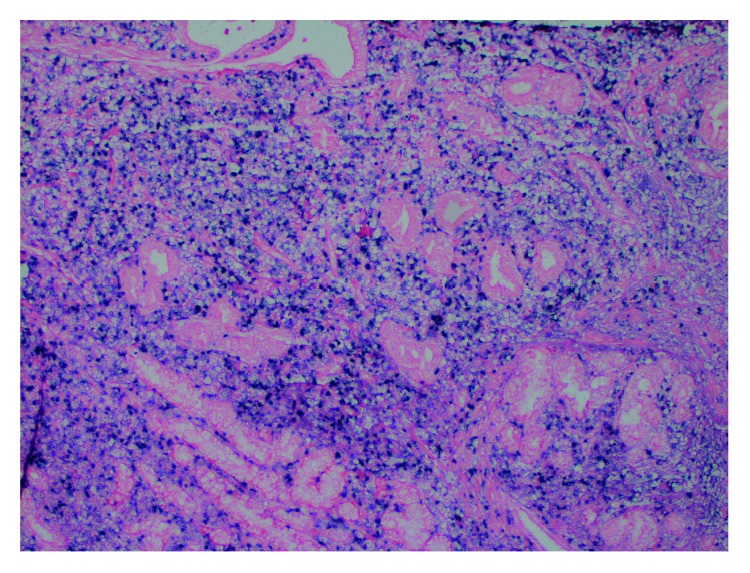
40x of the EBER-ISH stain on gastric biopsy demonstrating PBL with extensive involvement by EBV.

EBV is a common virus with pathogenic and oncogenic potential, a characteristic magnified in compromised immune systems [2]. Here, we describe a previously healthy patient presenting with EBV primary infection complicated by PBL and HLH resulting in multiorgan failure. The EBV virus was found in both bone marrow and gastric biopsy samples, implicating the important role it played in the pathogenesis of both processes (Figures 3 and 4). This report explores the relationship between EBV, immunity, and malignancy. In a healthy male, could his unique presentation be a manifestation of an underlying primary immunodeficient state?

In patients with normal immunity, EBV affects B cells but lies dormant until triggered by cellular stress. As the virus reproduces, it is recognized by cytotoxic immune cells and subsequently suppressed [2]. In patients with abnormalities in T and NK cell function, this can lead to inefficient immune cytotoxic activity, causing persistent immune activation and ultimately resulting in clinical HLH [2]. EBV is a well-recognized trigger for HLH, and a complicated clinical course of EBV primary infection should prompt an evaluation for immunodeficiencies. Studies conducted in post-stem cell transplant patients showed that chronically elevated EBV loads were associated with simultaneous T-cell activation and T-cell exhaustion suggesting that both an upregulated and a functional T-cell response are required to control EBV infection [3]. In both X-linked lymphoproliferative disorder (XLP) and X-linked inhibitor of apoptosis deficiency (XIAD), genetic mutations in T and NK cells impair cell-mediated immunity and lead to EBV proliferation [2, 4]. Even in chronic active EBV (CAEBV), an entity characterized by patients with persistent EBV infection and lack of known immunodeficiency, we are understanding more that it is likely due to lesser known B-cell mutations [5].

EBV is also recognized for its oncogenic potential. PBL is an aggressive B-cell malignancy of plasmablasts strongly associated with HIV. In affected patients, up to two thirds are HIV positive; however, among the HIV negative, fifty percent are positive for EBER [6, 7]. There have been no data to discuss the role of primary immunodeficiency or EBV in the pathogenesis of PBL, but EBV-associated lymphomas are well characterized. The myc rearrangement, the genetic hallmark of EBV-associated Burkitt's lymphoma, is also the most common cytogenetic finding in PBL [7]. Mutations in myc and its regulator, PRDM1/Blimp-1, can produce the PBL phenotype, and loss of function in this pathway was recently shown to produce more phenotypically aggressive diffuse large B-cell lymphomas [8, 9]. Nearly, all primary immunodeficiencies that are associated with EBV-driven malignancies are related to B, T, and NK cell function [10]. T and NK cells clear virally infected B cells but are also vitally important in clearing malignant cells [10]. EBV presence and viral load has even been studied as a potential prognostic marker of HIV-associated lymphomas [11]. Prognosis for PBL has been historically poor, with expected survival of less than 6 months in untreated patients [7]. Standard lymphoma regimens are most commonly followed such as CHOP (cyclophosphamide, doxorubicin, vincristine, and prednisone) or EPOCH (etoposide, cyclophosphamide, doxorubicin, vincristine, and prednisone); however, no standard of care exists [7]. Other immunomodulatory agents such as bortezomib and lenalidomide have had limited success and should be considered on an individual basis [12].

For our patient, initial workup for immunodeficiency was unrevealing. His HIV antibody and viral load were both negative repeatedly, and he had a normal karyotype. Further workup was not pursued as his acute illness worsened, but testing would have included B-cell phenotyping and genetic evaluation for common T, B, and NK cell mutations such as XLP (X-linked lymphoproliferative syndrome) and XIA (X-linked agammaglobulinemia), with a more targeted panel if those are negative. Patients with diagnosed genetic disorders should be considered for bone marrow transplant. This case illustrates the difficulties in diagnosing and managing the complications of EBV primary infection. It is imperative to consider an underlying primary immunodeficiency in patients with rare complications from EBV infection and to be aggressive about characterizing the full extent of immune dysregulation.
